# Functional Expression of NMDA Receptors in SH-SY5Y Neuroblastoma Cells Following Long-Term RA/BDNF-Induced Differentiation

**DOI:** 10.3390/neurosci6020047

**Published:** 2025-05-26

**Authors:** Ya-Jean Wang, Yun-Hsiang Chen, Eric Hwang, Che-Jui Yeh, You-Xuan Liu, Hwei-Hsien Chen, Sheng-Nan Wu

**Affiliations:** 1 Department of Senior Services Industry Management, Minghsin University of Science and Technology, Hsinchu 30401, Taiwan; 2 Department of Life Science, Fu-Jen Catholic University, New Taipei City 242062, Taiwan; 125648@mail.fju.edu.tw; 3 Center for Neuropsychiatric Research, National Health Research Institutes, Miaoli 35053, Taiwan; hwei@nhri.edu.tw; 4 Institute of Molecular Medicine and Bioengineering, National Yang Ming Chiao Tung University, Hsinchu 300093, Taiwan; hwangeric@nycu.edu.tw (E.H.); kevin09955159@gmail.com (C.-J.Y.); 5 Department of Biological Science and Technology, National Yang Ming Chiao Tung University, Hsinchu 300093, Taiwan; lyx0714.bt12@nycu.edu.tw; 6 Institute of Bioinformatics and Systems Biology, National Yang Ming Chiao Tung University, Hsinchu 300093, Taiwan; 7 Institute of Neuroscience, National Chengchi University, Taipei 116011, Taiwan; 8 Institute of Basic Medical Sciences, National Cheng Kung University Medical College, Tainan 70101, Taiwan; snwu@mail.ncku.edu.tw; 9 Department of Medical Research and Education, An Nan Hospital, China Medical University Hospital, China Medical University, Tainan 709204, Taiwan

**Keywords:** SH-SY5Y cells, differentiation, evoked action potential, NMDA receptor-mediated current, atorvastatin

## Abstract

SH-SY5Y neuroblastoma cells can be effectively differentiated into a neuronal phenotype using retinoic acid (RA) and brain-derived neurotrophic factor (BDNF), making them a valuable in vitro model for studying neuronal differentiation. This study aimed to investigate the electrophysiological properties of SH-SY5Y cells following prolonged differentiation, with a focus on membrane characteristics, evoked action potentials, and the functionality of cellular components such as N-methyl-D-aspartate (NMDA) receptor. Whole-cell patch-clamp recordings were employed to evaluate ionic currents and action potentials in embryonic mouse cortical neurons (mCNs) and in both differentiated and undifferentiated SH-SY5Y neuroblastoma cells. Differentiated SH-SY5Y cells exhibited neurite outgrowth, evoked action potential firing, and functional NMDA receptor-mediated currents. Notably, atorvastatin significantly modulated the duration and firing of action potentials as well as NMDA receptor-mediated currents in differentiated SH-SY5Y cells. These findings highlight that neuronally differentiated SH-SY5Y cells expressing functional NMDA receptor-mediated currents serve as a robust and convenient model for investigating the molecular mechanisms of NMDA receptor function and for screening pharmacological agents targeting these receptors.

## 1. Introduction

The SH-SY5Y human neuroblastoma cell line, a thrice-cloned subline of SK-N-SH, originates from the bone marrow biopsy of a 4-year-old female patient. Recognized as a well-established neuronal model [1,2,3,4,5], SH-SY5Y cells are extensively utilized in studies of neurodegenerative diseases, such as Parkinson’s and Alzheimer’s diseases, as well as neurotoxicity [6,7,8,9,10,11]. It is important to note that neuroblastoma, the origin of SH-SY5Y cells, is not a central nervous system (CNS) tumor. However, despite this, SH-SY5Y cells are widely used as a neuronal differentiation model due to their ability to differentiate into neuron-like cells under appropriate conditions. Due to their human origin, catecholaminergic neuronal properties, and ease of maintenance, SH-SY5Y cells are frequently chosen for research on neuronal function. Through various differentiation protocols, these cells can acquire a phenotype resembling human dopaminergic neurons. Recent studies have demonstrated that treatment with all-trans retinoic acid (RA) induces differentiation in SH-SY5Y cells, resulting in the formation and elongation of neurites and the expression of neuron-specific enzymes, neurotransmitters, and receptors. Moreover, RA-treated SH-SY5Y cells exhibit altered ion channel profiles, leading to increased membrane excitability, making them a valuable model for studying neuronal physiology and pathology [12].

A recent study introduced a simplified, neurotrophin-free method for differentiating and sustaining SH-SY5Y cells in long-term culture, demonstrating the gradual development of an ageing phenotype [13]. To induce neuronal differentiation, SH-SY5Y cells at 70% confluence were exposed to 10 μM retinoic acid, while the serum concentration was reduced to 1%. Additionally, the culture medium was supplemented with 18 μM 5-fluorodeoxyuridine (5-FDU) to inhibit cell proliferation and 2% serum replacement (SR-2; Sigma UK) to support viability. Long-term culture under these conditions not only triggered neuronal action potentials and sodium currents but also led to the accumulation of oxidative damage markers, which have previously been used to characterize the ageing phenotype in primary neurons in vitro. Interestingly, previous studies have indicated that NMDA receptors are mainly expressed in neurons of the central nervous system. Previous research has shown that retinoic acid (RA) and brain-derived neurotrophic factor (BDNF) together induce the expression of NR1, NR2C, and NR2D mRNA in SH-SY5Y cells, while NR2A and NR2B expression is nearly undetectable [14]. These results suggest that RA and BDNF may selectively regulate the expression of NMDA receptor subunits. The increase in NR2C and NR2D expression could potentially impact neurodifferentiation or synaptic plasticity, and these changes in receptor subunits may be related to neurodevelopment or neuroprotective effects. NR2A and NR2B are typically the more common NMDA receptor subunits and are primarily associated with synaptic transmission and learning and memory functions. In contrast, NR2C and NR2D are less commonly expressed in the adult brain, but their expression may be elevated during neurodevelopment or in the repair process following neural injury. Therefore, the role of RA and BDNF in neurodifferentiation may be linked to their promotion of specific NMDA receptor subunit expression, which could help facilitate neuronal growth, synaptic connectivity, and the recovery of neural function. Nevertheless, it remains unclear whether RA and BDNF-treated SH-SY5Y cells can exhibit functional NMDA receptor-mediated currents or evoked action potential. This study aimed to compare RA/BDNF-treated human neuroblastoma SH-SY5Y cells with untreated controls, exploring their potential as an in vitro model to investigate the characteristics of NMDA receptor-mediated currents and action potentials.

Atorvastatin is a synthetic, lipophilic statin widely used to lower blood cholesterol levels and is generally considered safe and well tolerated [15]. Several preclinical studies have demonstrated its neuroprotective effects in various models of neuropathology, including traumatic brain injury, NMDA receptor-mediated seizures, and β-amyloid-induced neurotoxicity [16,17,18]. Moreover, clinical evidence has shown an association between statin use and a reduced risk of depression and anxiety [19]. These findings suggest that atorvastatin may exert antidepressant-like effects, possibly through inhibition of NMDA receptor activity. Previous studies also reported that atorvastatin can attenuate glutamate-induced intracellular calcium elevation, further supporting its potential role in modulating NMDA receptor function. Based on these observations, we hypothesized that atorvastatin may modulate NMDA receptor-mediated currents in the differentiated SH-SY5Y cell model established in this study.

To investigate the electrophysiological properties of SH-SY5Y cells after prolonged differentiation, this study examined membrane characteristics, evoked action potentials, and the functionality of cellular components such as NMDA receptors. We found that prolonged differentiation of SH-SY5Y cells with RA and BDNF enabled the cells to generate action potentials in response to depolarizing currents and to express functional NMDA receptor-mediated currents. The neuron-like characteristics of these differentiated cells provide a robust and convenient model for studying the molecular mechanisms of NMDA receptor function and for screening pharmacological agents targeting these receptors. Interestingly, this study demonstrated that atorvastatin inhibited NMDA receptor-mediated currents and reduced neuronal action potential activity.

## 2. Materials and Methods

### 2.1. Reagents and Solutions

The following reagents were used in the experiments: atorvastatin, tetrodotoxin (TTX), MK-801, NMDA, glycine, tetraethylammonium chloride, CNQX, picrotoxin, retinoic acid (RA), brain-derived neurotrophic factor (BDNF), and papain, all sourced from Sigma-Aldrich (St. Louis, MO, USA), with TTX additionally provided by BD Bioscience. For NMDA-mediated current recordings, the bath solution contained 140 mM NaCl; 5 mM KCl; 1.8 mM CaCl_2_; 10 mM glucose; and 10 mM HEPES, adjusted to pH 7.4, and the patch pipette solution consisted of 140 mM K-gluconate; 4 mM NaCl; 0.5 mM CaCl_2_; 5 mM EGTA; 0.5 mM Na3GTP; and 10 mM HEPES, with a pH of 7.2. For action potential recordings, the patch solution included 145 mM KCl; 1 mM MgCl_2_; 0.1 mM EGTA; and 5.5 mM HEPES, adjusted to pH 7.2, and the chamber solution was made up of 136.5 mM NaCl; 5.4 mM KCl; 1.8 mM CaCl_2_; 0.53 mM MgCl_2_; 5.5 mM glucose; and 5.5 mM HEPES, with a pH of 7.4.

### 2.2. Preparation of Cells

SH-SY5Y cells were cultured at 37 °C with 5% CO_2_ in Dulbecco’s modified Eagle’s medium (DMEM) containing 15% fetal bovine serum (FBS; Gibco Life Technologies, Paisley, UK), 1% antibiotic solution (10,000 U/mL penicillin and 10,000 µg/mL streptomycin, Gibco), 1% non-essential amino acids (NEAA, Gibco Life Technologies, Paisley, UK ), and 1% sodium pyruvate (Gibco Life Technologies, Paisley, UK). The cultures were passaged before reaching confluence.

For differentiation, SH-SY5Y cells were seeded on fresh culture dishes or coverslips in basic growth medium at 40–50% confluency on day 1. On day 2, the medium was replaced with differentiation medium, which consisted of DMEM supplemented with 2 mM L-glutamine, 15% FBS, 100 U/mL penicillin, 100 µg/mL streptomycin, and 25 µM all-trans retinoic acid (RA). On day 4 (d4), the medium was refreshed with differentiation medium containing 50 µM all-trans RA. From days 6 to 20 (d6–d20), the medium was replaced with differentiation medium supplemented with 50 ng/mL BDNF and excluding fetal bovine serum. By days 10 to 20, the cells exhibited neuron-like morphology, including extended neurite outgrowth. Patch-clamp experiments were performed between days 15 (d15) and 20 (d20).

The protocol used to culture the embryonic mouse cortical neurons (mCNs) was as follows: All animal experiments were conducted in accordance with protocols approved by the Institutional Animal Care and Use Committee of the National Health Research Institutes (NHRI) (Approval number:109042-A). The cerebral cortex was dissected from embryonic day 18 (E18) ICR mouse embryos obtained from BioLASCO Taiwan Co., Ltd. (Taipei City, Taiwan). The isolated tissue was enzymatically and mechanically dissociated into single cells, followed by three PBS washes before resuspension in minimal essential medium. Primary cortical neurons were seeded in vitro on day 0 (DIV0) in neurobasal medium supplemented with 1% B27. To limit the proliferation of non-neuronal cells, 5 μM cytosine 1-β-D-arabinofuranoside (Ara-C) was added on DIV3 and maintained for 24 h. Thereafter, half of the culture medium was refreshed every three days using a 1:1 mixture of neurobasal and glutamine-containing media.

### 2.3. Electrophysiological Data Testing and Analysis

Action potential (AP) recordings from primary mCN neurons or SH-SY5Y cells were performed using the whole-cell patch-clamp technique. Membrane currents were recorded using a current-clamp protocol controlled by EPC 10 patch clamp amplifier and PatchMaster software (Version 1.3) (HEKA Elektronic, Lambrecht, Germany). The analog signals were filtered at 1 or 3 kHz, digitized at 10 kHz, and stored on a hard disk. Recording pipettes were fabricated from thin-walled capillary tubes (Kimax-51 #34500, Kimble, New Taipei City, Taiwan), with resistances ranging from 3 to 5 MΩ when immersed in the internal solutions. The junction potential was nulled before sealing the pipette. Evoked AP firing was triggered by holding the membrane potential at −70 mV and applying a positive current injection to depolarize the cell.

To assess NMDA receptor-mediated currents, SH-SY5Y cells were held at 0 mV and subjected to depolarizing pulses from −70 mV to +50 mV for 300 ms, with 10 mV incremental voltage steps applied. The bath solution included CNQX (10 μM) and picrotoxin (10 μM) to isolate NMDA receptor-mediated responses. Recordings were taken from SH-SY5Y cells between differentiation days 15 and 20 (d15−d20). To test the effect of atorvastatin on NMDA receptor-mediated currents, the holding potential was maintained at 0 mV, and a clamp pulse step to −70 mV was applied for 300 ms. NMDA (100 μM) and glycine (10 μM) were then co-applied to induce NMDA receptor activation, and the response was compared to the control (NMDA/glycine alone) within the same cell.

### 2.4. Statistical Analyses

All statistical analyses were performed using pCLAMP 9.0 software (Molecular Devices, San Jose, CA, USA), OriginPro 7.5 software (Microcal Software, Inc., Northampton, MA, USA), SigmaPlot 7.0 software (SPSS Inc., Chicago, IL, USA), or custom-made macros in Excel 2003 (Microsoft Corp., Redmond, WA, USA). Data are expressed as mean ± SEM, with sample sizes (n) representing the number of recorded cells. Comparisons of membrane ionic currents and action potential firing parameters between groups were primarily conducted using the Student’s *t*-test (paired or unpaired). For multiple-group comparisons, one-way ANOVA followed by Tukey’s post hoc test was applied. Statistical significance was set at *p* < 0.05.

## 3. Results

### 3.1. Differentiated SH-SY5Y Cells Treated with RA/BDNF Were Viable in Long-Term Culture and Exhibited Significant Morphological Changes and Pronounced Neurite Outgrowth

**Figure 1:**
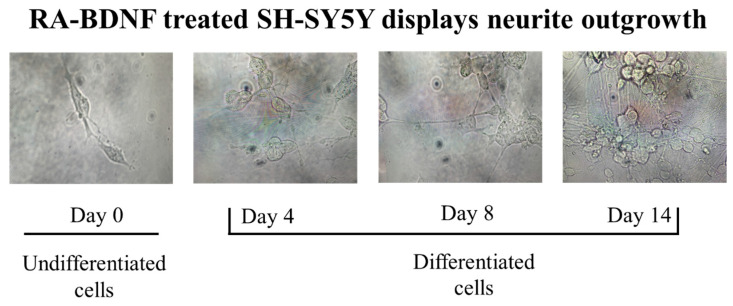
Phenotypic characterization of RA-BDNF-differentiated SH-SY5Y cells. At the differentiation treatment, we found that differentiated cells showed significant neurite outgrowth compared with undifferentiated cells. Phase-contrast photomicrographs of SH-SY5Y cells after 0, 4, 8, and 14 days of differentiation.

To determine whether SH-SY5Y cells could survive and differentiate in long-term culture without fetal bovine serum support after treatment with retinoic acid (RA) and brain-derived neurotrophic factor (BDNF), we observed changes in cell morphology and survival status over a 20-day period post-treatment. As shown in Figure 1, day 0 represents cells cultured in DMEM medium containing 15% serum, following the conditions reported in a previous study, without RA and BDNF [20]. At this stage, the cells exhibited a larger cell body with an elongated shape and no apparent dendritic spines. From day 1 to day 2, the cells were cultured in DMEM medium containing 15% serum and 25 µM RA. Subsequently, from day 3 to day 4, the cells were transferred to DMEM medium containing 15% serum and 50 µM RA for continued culture. By day 4, the cell morphology began to change, with the cell bodies becoming smaller and adopting a rounded appearance, while small dendritic spines started to emerge. For BDNF-induced differentiation of SH-SY5Y cells, the cells were then cultured in serum-free DMEM medium containing 50 ng/mL BDNF for an additional 16 days, up to day 20. Interestingly, from day 8 onwards, a significant number of elongated neurites were observed, forming connections between cells. By day 14, the neurites became densely distributed among the cells, with the cell bodies largely clustered together to form colonies. These changes clearly indicate that after more than 8 days of differentiation, the cells had developed extensive neurite outgrowth, forming an interconnected network (*n* = 5).

### 3.2. SH-SY5Y Cells Differentiated with RA/BDNF Treatment Exhibited Evoked Action Potential

**Figure 2:**
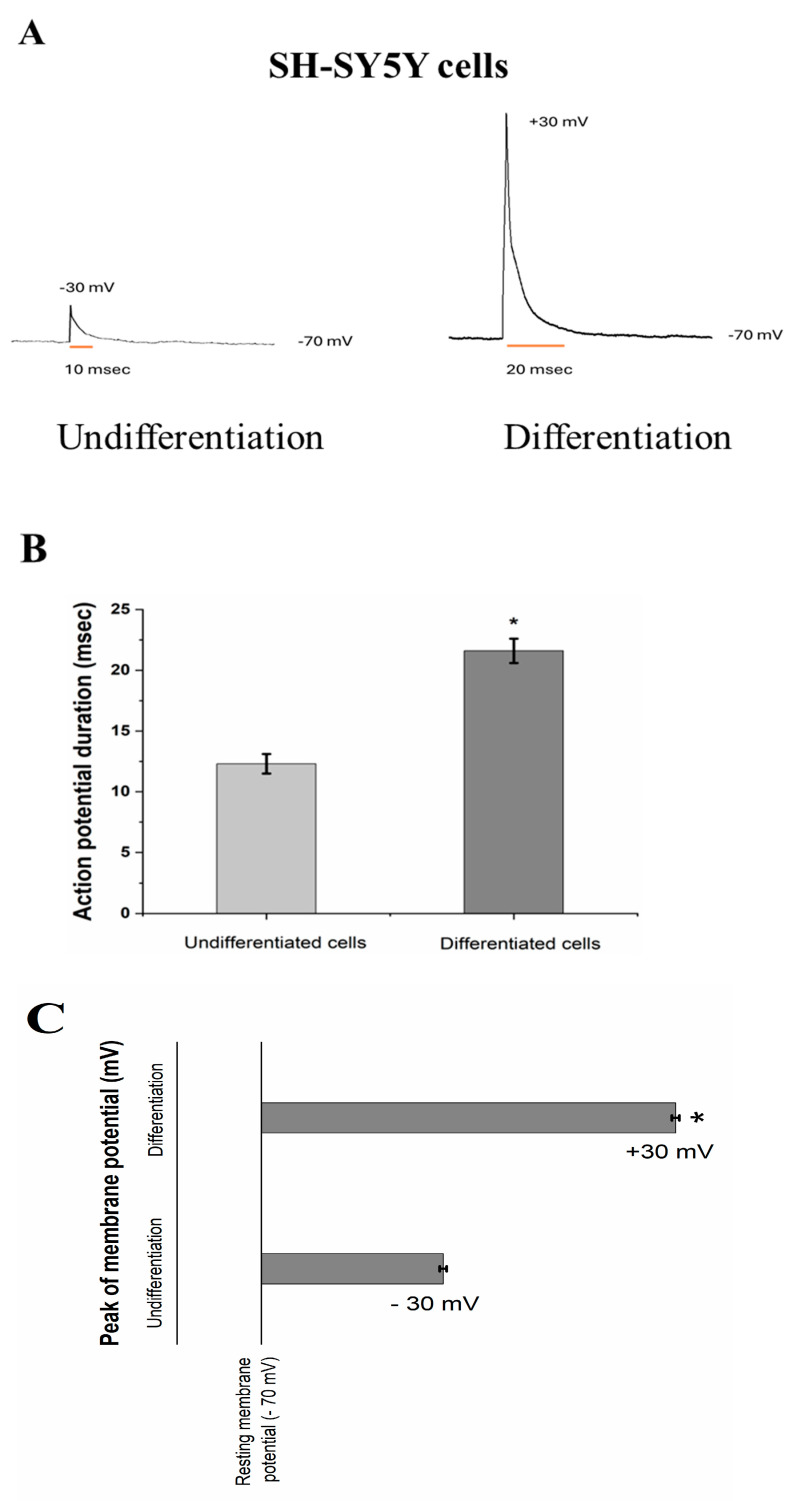
Representative whole−cell patch−clamp recordings show action potential firing in undifferentiated cells and differentiated cells. Panel (**A**) displays action potentials induced by current injection (1 ms, 200 pA) in undifferentiated cells and differentiated cells, panel (**B**) illustrates the duration of these action potentials in undifferentiated cells and differentiated cells, and panel (**C**) illustrates the peak of these action potentials in undifferentiated cells and differentiated cells. * Significantly different from undifferentiation (*p* < 0.01).

In addition to the effects of RA/BDNF treatment on neuronal neurite outgrowth and the formation of interconnected networks, the action potential (AP) is also an important indicator of neuronal function and maturity. In this study, four to five cells from each group of SH-SY5Y cells, before and after RA/BDNF treatment, were randomly selected and assessed using single-cell current-clamp testing to evaluate the effects on action potential and investigate differences in neuronal maturation levels. We performed whole-cell patch-clamp electrophysiological recordings on cultures of 14- to 20-day-old cells to evaluate the membrane properties. To confirm the ability of differentiated cells to generate neuronal action potential, current-clamp recordings showed that holding the membrane potential at −70 mV and applying a positive current stimulus (200 pA; 1 ms duration) evoked a single action potential [21]. The action potential had a peak of approximately +30 ± 11 mV and a duration of about 20 ± 5.6 ms. In contrast, undifferentiated cells failed to generate neuron-like action potentials, even when larger positive current stimuli were applied (Figure 2A). Figure 2B,C illustrate the duration and peak amplitude of action potentials in undifferentiated and differentiated cells. The results show that both the duration and amplitude of action potential significantly increased after differentiation (*p* < 0.05, *n* = 4–5). These results demonstrated that cells not treated with RA/BDNF exhibited extremely minimal changes in membrane potential in response to current stimulation. Taken together, these findings demonstrate that the differentiated cells exhibited functional neuronal characteristics. The appearance of action potential responses suggests that the differentiated neurons were more mature and likely possessed more active ion channels or cell surface receptors, which played a role in the generation of action potentials.

### 3.3. Long-Term RA/BDNF-Treated SH-SY5Y Cells Displayed NMDA Receptor-Mediated Currents

**Figure 3:**
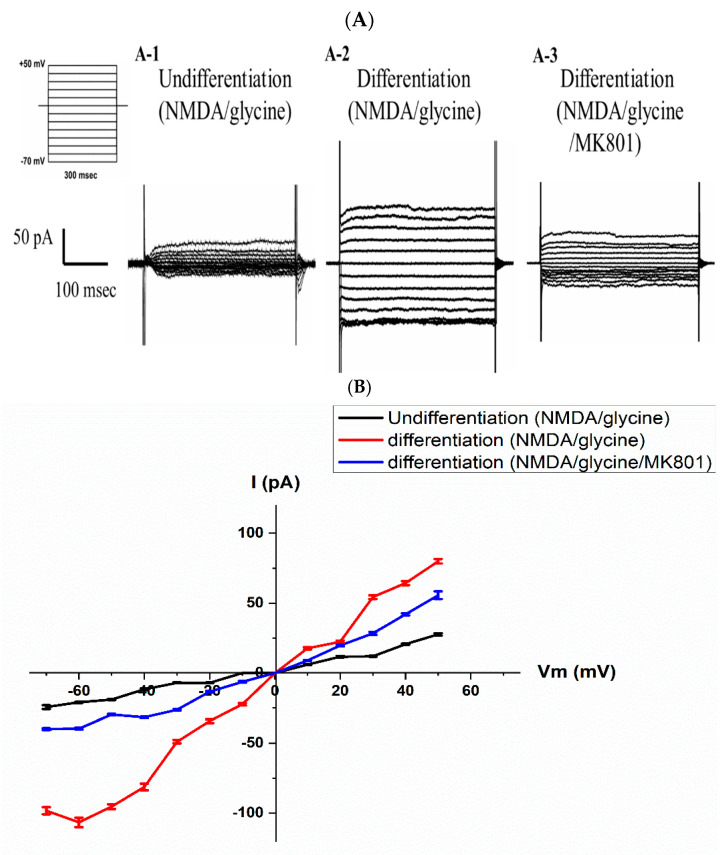
Electrophysiological current responses in undifferentiated and RA−BDNF−differentiated cells. Representative whole−cell current traces recorded from undifferentiated cells (**A-1**) and differentiated (**A-2**,**A-3**) cells using the voltage protocols indicated at the top of the panels. Depolarizing voltage steps were applied with an increment of 10 mV each for 300 ms, starting from −70 mV to +50 mV, holding the cells at 0 mV. (**A**) Representative traces showing agonist-evoked NMDAR currents elicited at a series of membrane potentials from −70 mV to +50 mV (in 10 mV increments) in the absence (**A-1**) of NMDA/glycine, presence (**A-2**) of NMDA/glycine, and presence (**A-3**) of NMDA/glycine/MK801 in RA−BDN−treated SH-SY5Y cells. (**B**) Figure shows the voltage dependence of the current evoked by NMDA/glycine in differentiated cells.

Brain-derived neurotrophic factor (BDNF) plays a crucial role in maintaining synaptic plasticity involved in learning and memory. Previous literature has indicated that acute stimulation of hippocampal neurons with BDNF differentially upregulates the protein levels of NR1, NR2A, and NR2B NMDA receptor subunits. Additionally, BDNF increases the abundance of NMDA receptors and their delivery to the plasma membrane, thereby upregulating receptor activity in cultured hippocampal neurons [22]. It is well known that SH-SY5Y cells express endogenous NMDA receptors at the protein level in the cytosol [23], but there is currently no evidence demonstrating functional NMDA receptor activity on the plasma membrane of SH-SY5Y cells. Therefore, this study also investigated whether SH-SY5Y cells, following long-term differentiation induced by BDNF, expressed functional NMDA receptors. Voltage-clamp techniques were employed to determine whether long-term RA/BDNF-induced differentiation led to the expression of NMDA receptor-mediated currents in these cells. We compared NMDA receptor-mediated currents between undifferentiated and RA/BDNF-differentiated SH-SY5Y cells to assess the impact of RA/BDNF treatment on NMDA receptor function. NMDA receptor activation requires the simultaneous presence of glutamate and glycine, which act as co-agonists. When both molecules bind to their respective sites on the NMDA receptor, it undergoes a conformational change, opening the ion channel and allowing the flow of ions, resulting in the generation of an NMDA-mediated current. This co-activation property distinguishes NMDA receptors from other glutamate receptors, such as AMPA receptors, where only glutamate binding is sufficient for activation. In summary, NMDA acts as the primary agonist, directly activating the receptor and opening the ion channel, while glycine serves as a necessary co-agonist, enabling full activation of the NMDA receptor and the subsequent functional NMDA-mediated current. When measuring NMDA receptor-mediated currents, we set the whole-cell recording holding potential to 0 mV to stabilize the measurement of NMDA receptor current responses. This approach helped minimize interference from other ion channels, allowing us to focus on NMDA receptor currents. Subsequently, a voltage range from −70 mV to +50 mV was applied to stimulate NMDA receptor-mediated current responses and determine NMDA receptor activity at different voltages. As shown in Figure 3(A-1), representative whole-cell current recordings from undifferentiated cells show that NMDA/glycine-induced NMDA receptor currents were not prominent across a range of membrane potentials (from −70 mV to +50 mV in 10 mV increments). In contrast, Figure 3(A-2) presents representative whole-cell current recordings from differentiated cells, demonstrating a significant increase in NMDA/glycine-induced NMDA receptor currents within the same membrane potential range. The current amplitude in differentiated cells markedly increased from approximately 50 pA in undifferentiated cells to around 150 pA, reflecting nearly threefold enhancement. However, when differentiated SH-SY5Y cells were treated with MK-801 (an NMDA receptor inhibitor), these NMDA/glycine-induced NMDA receptor currents were significantly suppressed (Figure 3(A-3)). This further indicates that the NMDA/glycine-induced increase in current observed in differentiated SH-SY5Y cells was mediated by NMDA receptor activity. Figure 3B presents the *I*-*V* relationship curve, comparing the currents evoked by NMDA/glycine and NMDA/glycine/MK-801 in undifferentiated and differentiated SH-SY5Y cells. The results indicate that differentiated SH-SY5Y cells exhibited functional NMDA receptor-mediated currents, which were significantly inhibited by MK-801 (*p* < 0.05, *n* = 4). The emergence of NMDA receptor-mediated currents was observed starting from day 14 and persisted through day 20, reflecting the stable establishment of functional NMDA receptor activity over the course of differentiation.

### 3.4. Pharmacological Characteristics of NMDA Receptor-Mediated Action Potential Firing in Differentiated SH-SY5Y Cells

**Figure 4:**
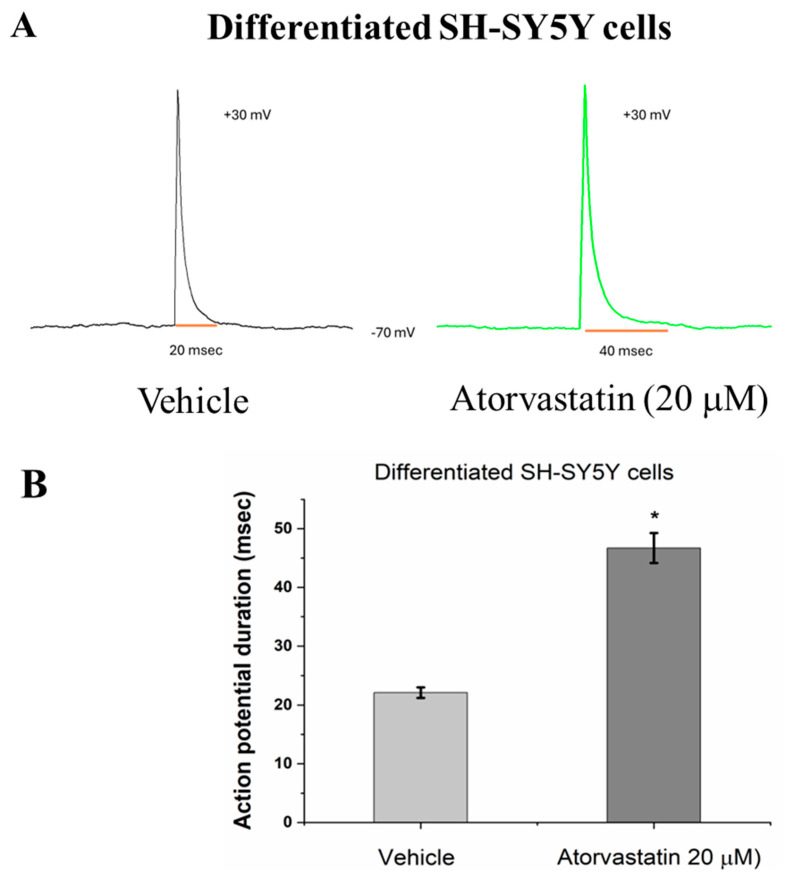

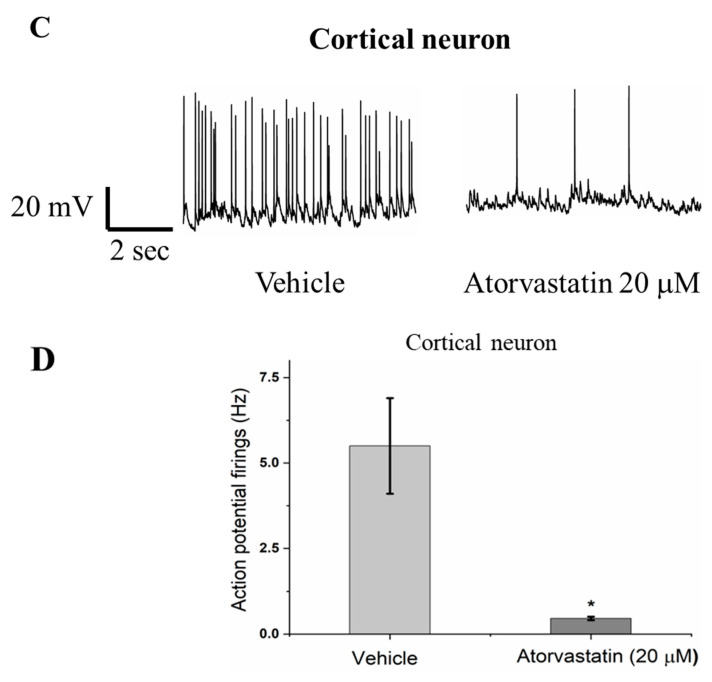
Representative whole-cell patch-clamp recordings show action potential firing in undifferentiated and differentiated cells. Panel (**A**) displays action potentials induced by current injection in differentiated cells, panel (**B**) illustrates the effect of atorvastatin on the duration of these action potentials, and panels (**C**,**D**) demonstrates the impact of atorvastatin on the action potential firing rate induced by NMDA/glycine in cortical neurons. * Significantly different from Vehicle (*p* < 0.01).

The effects of pharmacological agents on action potential generation in differentiated SH-SY5Y cells can be assessed through electrophysiological recordings to evaluate their functional and pharmacological properties. In this study, we aimed to determine whether differentiated SH-SY5Y cells were suitable for electrophysiological recordings to assess their pharmacological properties (including changes in action potentials), and we compared undifferentiated and differentiated SH-SY5Y cells. We treated these cells with atorvastatin and found that atorvastatin increased the duration of action potentials (from 22.1 ± 0.9 ms to 46.7 ± 2.6 ms) (as shown in Figure 4A,B, *p* < 0.05, *n* = 4). However, the effect of atorvastatin on the amplitude of action potentials in differentiated SH-SY5Y cells was not significant (from +29 ± 0.8 mV to +28 ± 0.9 mV). Since neuronal excitability is associated with action potential duration, we further examined the effect of atorvastatin on action potential firing using primary cortical neurons. As shown in Figure 4, atorvastatin (20 µM) significantly reduced the action potential firing rate. For example, the results in Figure 4D indicate that the firing rate decreased from 5.5 ± 0.17 Hz to 0.46 ± 0.04 Hz (*p* < 0.05, *n* = 4). However, atorvastatin had no significant effect on action potential amplitude (from 52 ± 12 mV to 55 ± 5 mV).

### 3.5. Pharmacological Characteristic of NMDA Receptor-Mediated Current in Differentiated SH-SY5Y Cells

**Figure 5:**
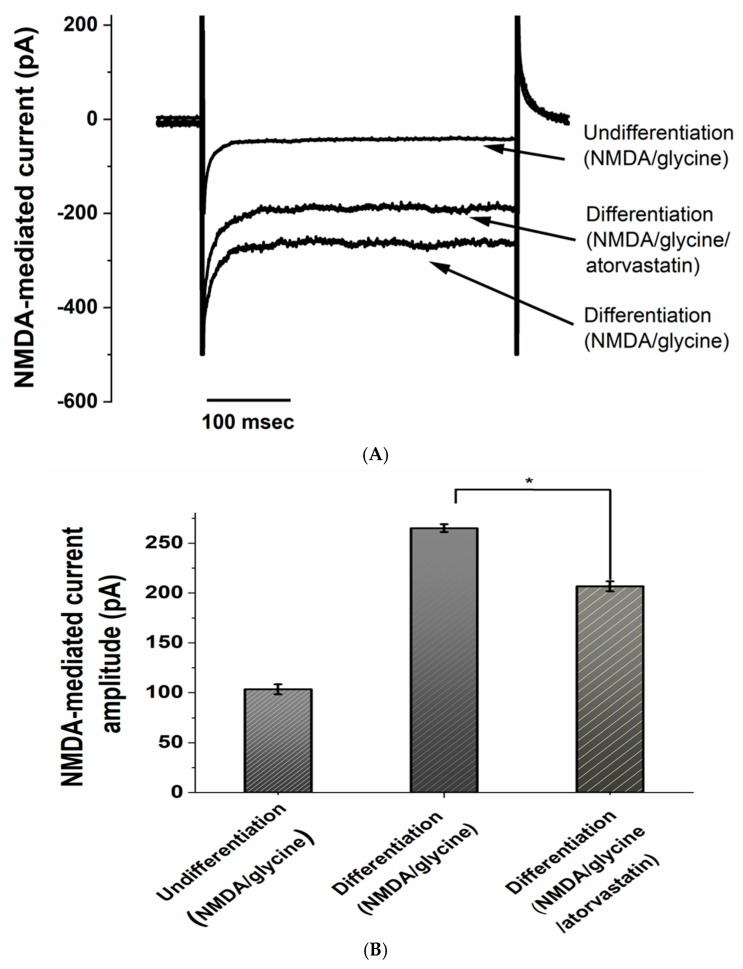
Inhibition of NMDA/glycine-evoked currents in differentiated SH-SY5Y cells by atorvastatin. (**A**) All cells were held at 0 mV in an external solution without MgCl_2_. The cell membrane potential was stepped from 0 mV to −70 mV for 300 ms, and current traces were recorded after treatment with NMDA/glycine. (**B**) Histogram showing the peak amplitudes of the inward currents after treatment with atorvastatin (20 μM). * Significantly different from undifferentiation (*p* < 0.01).

As shown in Figure 5, NMDA/glycine-induced currents were not prominent in undifferentiated SH-SY5Y cells. However, in patch-clamp experiments, we observed a significant increase in NMDA/glycine-mediated current amplitude in RA-BDNF-differentiated cells. Moreover, this current amplitude was significantly inhibited by atorvastatin. For example, in undifferentiated cells, the NMDA/glycine-induced current amplitude was 103 ± 5.1 pA, whereas in differentiated cells, it increased to 265 ± 4.4 pA. Interestingly, atorvastatin (20 μM) significantly inhibited the NMDA/glycine-induced current amplitude in differentiated cells, reducing it from 265 ± 4.4 pA to 206 ± 5.2 pA, with statistical significance (*p* ≤ 0.05, *n* = 4).

## 4. Discussion

The neuroblastoma cell line SH-SY5Y is widely used in neurodegenerative disease research due to its human protein expression and neuronal phenotype [24,25]. Retinoic acid (RA) promotes the upregulation of neuronal marker proteins and enhances neurite outgrowth [26,27,28]. The addition of growth factors further reduces cell proliferation, enhances neurite extension, and increases the expression of neuron-specific marker protein [6,20,29]. Therefore, while RA alone can induce differentiation, the subsequent addition of growth factors is necessary for the long-term survival of differentiated neurons. During the differentiation phase, serum concentration is typically reduced to 1% to inhibit cell proliferation and promote differentiation [30]. In this study, we reduced the serum concentration from 15% to 0% during the differentiation phase before transferring the cells to different culture conditions. Previous studies have reported that without the supplementation of neurotrophic factors such as nerve growth factor (NGF) or brain-derived neurotrophic factor (BDNF), these cultured cells exhibit higher cell death rates and lower expression of neuron-specific marker proteins [6,26]. In our study, we observed that cell viability did not significantly decline over a 20-day culture period, indicating that under RA/BDNF culture conditions, these cells can maintain survival within this timeframe.

Our study investigated the electrophysiological changes in SH-SY5Y human neuroblastoma cells following long-term differentiation induced by RA/BDNF treatment. After 14–20 days of culture, the morphology of these cells underwent significant changes, with an increase in membrane branching and a notable enhancement in intercellular connection density. Based on these observations, we used the patch-clamp technique to measure the electrophysiological properties of the cell membrane. The results showed that in the cell population with more branched projections, membrane electrical properties also changed, further highlighting the importance of neuronal morphology in influencing electrophysiological characteristics [31,32]. To verify whether these cells possessed the key feature of neurons—excitability—electrophysiological recordings were conducted. Our results demonstrated that after long-term RA/BDNF treatment, the cells were able to generate action potential in response to current stimulation (Figure 2). The characteristics of the action potential closely resembled those of typical neuronal action potential, with an action potential duration of approximately 20 ms and an action potential peak reaching around +30 mV. Taken together, these data confirm that the cells had differentiated into a functional neuronal phenotype, characterized by their excitability.

However, under our experimental conditions, the cells did not exhibit repetitive firing. Notably, recent studies have shown that with different long-term differentiation protocols, SH-SY5Y cells are capable of generating clear repetitive firing and expressing appropriate ion channels [13]. Our study speculated that the absence of repetitive firing may have been related to the incomplete maturation of various potassium ion channels, particularly Kv1.2, Kv3.1, and KCNQ2/3 (M-current), which are known to play a critical role in repetitive firing. Interestingly, these ion channels have been confirmed to be expressed in SH-SY5Y cell membranes [33,34,35]. However, our differentiation protocol may not have fully promoted the maturation of endogenous Kv1.2, Kv3.1, and KCNQ2/3 (M-current), leading to insufficient afterhyperpolarization (AHP) in the later phase of the action potential, thereby limiting the cells’ ability to exhibit repetitive firing. Future studies will further examine and compare the electrophysiological properties of SH-SY5Y cells before and after differentiation to more precisely evaluate the maturation of potassium ion channels. This will help optimize the differentiation protocol and enhance the applicability of this culture model, making it an ideal tool for generating high-purity neuronal cell cultures suitable for precise functional analyses.

P19 cells are murine cells (commonly used as substitutes for human CNS neurons), and previous literature has confirmed that differentiated P19 cells express various receptors and ion channels present in normal neurons, such as NMDA receptors, AKPA/kainate receptors, nicotine receptors, ergoline receptors, and calcium channels [36]. Interestingly, previous literature pointed out that SH-SY5Y human neuroblastoma cells, when differentiated by combined treatment with brain-derived neurotrophic factor (BDNF) and retinoic acid, clearly express mRNA for NR1, NR2C, and NR2D, with very weak expression of NR2A mRNA. Another report suggests that SH-SY5Y cells only express NR1 mRNA, regardless of differentiation state [37]. This discrepancy may be related to differences in PCR primers. Despite the controversy, no literature has reported whether SH-SY5Y cells express functional NMDA receptors after RA/BDNF differentiation. Therefore, this study compared the electrophysiological characteristics of undifferentiated and differentiated SH-SY5Y human neuroblastoma cells to examine whether prolonged culture time would lead to changes in NMDA receptor-mediated currents on the cell membrane. This study revealed, for the first time, that when the cells were cultured for 20 days, NMDA receptor-mediated currents were significantly affected (as shown in Figure 3). The electrophysiological properties of differentiated SH-SY5Y cells changed over prolonged culture, which was associated not only with an increase in NMDA receptor-mediated currents but also with changes in the cell membrane potential. These changes may work in concert to regulate the excitability of the cells. NMDA receptors are primarily expressed in neurons of the central nervous system. Previous studies have reported that after 5 days of RA/BDNF treatment, SH-SY5Y cells exhibited relatively high mRNA expression levels of NR1, NR2C, and NR2D subunits, while the expression of NR2A and NR2B was barely detectable [14]. Based on these findings, we speculate that the functional NMDA-mediated currents induced in our study after 14–20 days of RA/BDNF treatment were likely mediated by NMDA receptors containing NR2C and/or NR2D subunits. To validate the system as a tool for evaluating drug modulation, we tested whether atorvastatin affected the action potential duration (APD) and NMDA receptor-mediated currents in differentiated cells. These results were consistent with cortical neuron firing results (as shown in Figure 4). This differentiation system not only serves as an evaluation tool for these functions but also allows for the verification that atorvastatin has a property of reducing neuronal excitability.

The results of Figure 3 in the present study demonstrate that MK801 inhibited approximately 50% of the NMDA/glycine-induced inward currents. MK801 is a non-competitive NMDA receptor antagonist that requires receptor activation and channel opening to enter the ion channel and exert its blocking effect. Therefore, its inhibitory efficacy depends on the degree of receptor activation and the duration of channel opening; if receptor activation is infrequent, complete blockade may not be achieved. Furthermore, a relatively low concentration of MK801 or insufficient exposure time may also result in only partial inhibition of NMDA receptor-mediated inward currents. In addition, NMDA receptors are highly heterogeneous and are typically composed of different subunits, such as NR1, NR2A, and NR2B. Previous studies have indicated that the sensitivity of NMDA receptors to MK801 varies depending on their subunit composition, which may influence the blocking efficiency. Therefore, if multiple receptor subtypes coexist, some may exhibit reduced sensitivity to MK801, leading to partial inhibition. In this study, the observed inhibition rate of approximately 50% was consistent with previous reports [38], suggesting that MK801 cannot completely suppress all NMDA receptor-mediated currents. It is also noteworthy that the MK801 concentrations used in previous studies were generally higher than that employed in the present study, which could contribute to differences in blockade efficiency.

NMDA receptor activity plays a critical role in regulating neuronal excitability and the generation of action potentials. In this study, we demonstrated that atorvastatin significantly inhibited NMDA-mediated currents and reduced NMDA-dependent action potential firing, suggesting that it may decrease neuronal excitability through modulation of NMDA receptor function. Based on these findings, we propose that differentiated SH-SY5Y cells exhibiting NMDA receptor activity may serve as a potential in vitro model for antiepileptic drug screening. Beyond applications in epilepsy drug development, this cellular model may also be utilized to evaluate the neuroprotective or neurotoxic effects of compounds targeting glutamatergic neurotransmission. Finally, NMDA receptors play an important role in neurodegenerative diseases and cognitive processes. The novel neural cell model proposed in this study provides a more promising tool for the pharmacological and physiological research of NMDA receptors. This study confirms that RA/BDNF-differentiated SH-SY5Y cells are a novel model for studying functional NMDA receptors, capable of serving as an effective tool to test NMDA receptor function and neuroactive drugs (including action potentials), which contributes to a better understanding of their potential drug development and receptor functions in humans. Furthermore, such a cell model helps reduce the use of animal-derived cells.

## 5. Conclusions

Cell lines may develop heterogeneity over successive passages, which can potentially affect their responses to differentiation inducers. However, our study strictly limited all experiments to cells within 15 passages. No passage-dependent changes were observed in membrane electrophysiological properties, and the morphological and functional outcomes following differentiation were consistent. Therefore, future studies will continue to use cells within 15 passages to minimize variability between experimental batches. In addition, serum such as fetal bovine serum (FBS) contains various undefined growth and differentiation factors, and its composition can vary significantly between batches. This variability may influence differentiation efficiency, gene expression, drug responsiveness, and experimental reproducibility. These factors are especially critical in studies involving neural differentiation or receptor function analysis, where cells are particularly sensitive to environmental conditions. To improve the accuracy and consistency of future experiments, serum-free or chemically defined culture systems (such as neurobasal medium supplemented with B27) will be used in the experiments. Regarding the use and selection of serum, the same batch of serum will also be used in long-term experiments to reduce variability.

## Data Availability

The data supporting the findings of this study are not publicly available due to privacy and confidentiality considerations. Researchers interested in accessing the data may contact the corresponding author. Limited access may be granted under reasonable conditions and in accordance with ethical guidelines.
